# Barriers to infection prevention and control in long-term care/assisted living settings in British Columbia during the COVID-19 pandemic: a cross-sectional survey

**DOI:** 10.1186/s13756-023-01292-2

**Published:** 2023-08-30

**Authors:** Jocelyn A. Srigley, Brooke Cheng, Jun Chen Collet, Tara Donovan Towell, Guanghong Han, Dave Keen, Ka Wai Leung, Julie Mori, R. Ayesha Ali

**Affiliations:** 1 Faculty of Medicine, University of British Columbia, 317 - 2194 Health Sciences Mall, Vancouver, BC V6T 1Z3 Canada; 2 Department of Pathology and Laboratory Medicine, BC Children’s Hospital and BC Women’s Hospital and Health Centre, 4500 Oak St, Room 2J3, Vancouver, BC V6H 3N1 Canada; 3 Provincial Health Services Authority, 200-1333 W Broadway, Vancouver, BC V6H 4C1 Canada; 4 Fraser Health Authority, Suite 400, Central City Tower 13450 – 102nd Avenue, Surrey, BC V3T 0H1 Canada; 5 Interior Health Authority, 505 Doyle Ave, Kelowna, BC V1Y 0C5 Canada; 6 Department of Mathematics and Statistics, University of Guelph, 50 Stone Road East, Room 437 MacNaughton Building, Guelph, ON N1G 2W1 Canada

**Keywords:** Infection prevention and control, Long-term care, Survey, COVID-19

## Abstract

**Background:**

The COVID-19 pandemic disproportionately impacted long-term care and assisted living (LTC/AL) facilities in Canada, where infection prevention and control (IPAC) programs had been suboptimal. We aimed to identify barriers affecting healthcare workers’ (HCW) adherence to IPAC practices during the pandemic in British Columbia in LTC/AL compared to acute care settings.

**Methods:**

We conducted a web-based survey of direct care providers and IPAC professionals across BC from August to September 2021, focused on knowledge and attitudes toward IPAC within the context of the COVID-19 pandemic, and barriers that affected respondents’ abilities to follow IPAC practices throughout the pandemic.

**Results:**

The final analysis included 896 acute care respondents and 441 from LTC/AL. More LTC/AL respondents reported experiencing the following barriers: following IPAC guidance was of lower priority compared to other tasks (29.1% vs. 14.7%, FDR = 0.001) and not their responsibility (28.0% vs. 11.2%, FDR = 0.001); limited supplies for personal protective equipment (PPE) (49.0% vs. 33.6%, FDR = 0.001), hand hygiene products (42.2% vs. 28.8%, FDR = 0.001), and cleaning/disinfection products (44.1% vs. 30.3%, FDR = 0.001); deficits in IPAC leadership support (46.2% vs. 38.9%, FDR = 0.012), IPAC education and training (46.9% vs. 32.0%, FDR = 0.001), and patient care knowledge for managing COVID-19 infections (46.6% vs. 36.0%, FDR = 0.001).

**Conclusions:**

This survey found that barriers to HCWs’ adherence to IPAC practices during the COVID-19 pandemic were different in LTC/AL settings compared to acute care. Improvement efforts should focus on strengthening IPAC programs in LTC/AL, particularly enhanced IPAC staffing/leadership, increased training and education, and improving access to PPE, hand hygiene, and cleaning products.

**Supplementary Information:**

The online version contains supplementary material available at 10.1186/s13756-023-01292-2.

## Background

The COVID-19 pandemic has had widespread impacts on the Canadian health care system, but long-term care and assisted living (LTC/AL) facilities were disproportionately affected, particularly during the first two waves of COVID-19 [1–4]. As of May 2020, LTC residents accounted for 81% of reported COVID-19 deaths in Canada [5]. By February 2021, there had been COVID-19 outbreaks in over 2,500 LTC/AL facilities with over 78,000 residents and staff infected, representing approximately 10% of all COVID-19 cases in Canada [4]. At that point, there had been 14,739 deaths among LTC/AL residents, representing 69% of all COVID-19 deaths [4].

Prior to the pandemic, infection prevention and control (IPAC) programs in Canadian LTC/AL facilities were known to be suboptimal [6, 7]. Although the true incidence of outbreaks in LTC/AL facilities was not well documented, data from Canada [8] and elsewhere [9] suggest that outbreaks of respiratory viruses and other infections occurred commonly. Factors that may contribute to transmission of infections in these settings include frailty of residents, comorbid illnesses, physical infrastructure, limited resources, and staffing policies [10]. The emergence of the novel SARS-CoV-2 virus and rapidly evolving guidelines likely exacerbated the pre-existing challenges. Given the significant impact of COVID-19 and other healthcare-associated infections in LTC/AL, it is imperative to understand the barriers to implementing IPAC measures in order to better protect residents and healthcare workers (HCWs) in these settings. Our objective in this study was to examine the barriers to adherence with recommended COVID-19 IPAC practices among HCWs in British Columbia (BC) and to learn how barriers in LTC/AL differed from acute care settings.

## Methods

### Study Design

This project consisted of a cross-sectional, web-based open survey of IPAC knowledge, attitudes, and practices among HCWs in BC during the COVID-19 pandemic. The results are reported in accordance with the Checklist for Reporting Results of Internet E-Surveys (CHERRIES) [11].

### Setting and Population

Eligible respondents included HCWs working in acute care, long-term care/assisted living (LTC/AL), outpatient settings, pre-hospital care, and/or home care who provided direct patient care, as well as IPAC professionals who interacted with and educated direct care providers. Direct patient care was defined as working in the patient environment (e.g., entering patient rooms, face-to-face interaction with patients). This particular analysis focused on respondents from LTC/AL and compared to respondents from acute care. In BC, each health authority has a robust IPAC program to support acute care but IPAC programs for other settings are more variable, so acute care was chosen as the comparator. Participants had to work at one of the eight health authorities in BC as their primary work environment.

The secure online survey tool REDCap [12] was used to host and collect the survey responses from a convenience sample of HCWs who provided informed consent. Recruitment of participants occurred from August 11, 2021, to September 24, 2021 via staff newsletters and communications platforms of all health authorities in BC, as well as social media and public websites of health authorities and affiliated organizations. To incentivize participation, six participants were randomly chosen to each receive a $50 Amazon gift card. Contact information of participants who wished to be entered into the draw was not linked to survey responses. Following promotion of the survey on health authority social media channels in August 2021, we received a high volume of suspected spam entries. We temporarily closed the survey for four days and implemented additional measures to prevent spam responses.

### Data source

A survey was developed based on the Theoretical Domains Framework, [13] existing survey tools, [14] and expert opinion from the multidisciplinary study team (Supplementary Material). The survey consisted of 93 items distributed over 9 pages and included the following sections: (1) knowledge assessment section of COVID-19 IPAC practices for HCWs providing direct care; (2) potential barriers to HCW use of COVID-19 IPAC practices, organized into the categories of perception, guidance and communication, infrastructure, and front line work environment; (3) suggestions for how to overcome the barriers identified in the section above. A 5-point Likert scale was used by respondents to rank the importance of each barrier, and a free text option was provided for additional barriers to be documented.

The survey was piloted by a convenience sample of HCWs including both non-IPAC and IPAC professionals from various health authorities and healthcare settings, who provided feedback on the readability and content of the survey questions. Adaptive questioning was used and respondents were able to review and change their answers. The REDCap platform was not set up for randomization of items, completeness checks, determining unique site visitors, determining view rate, cookies, collecting IP addresses, or log file analysis.

### Statistical analysis

For analysis purposes, we excluded any responses that were incomplete, not fully submitted, or did not have a properly filled out consent. To minimize the potential impact of potential spam entries on one particular group, we aggregated responses from non-IPAC and IPAC professionals whenever questions were relevant to both groups. We conducted sensitivity analyses (data not shown) and found that responses before and after implementation of the additional measures were not statistically significantly different. Therefore, although there may be a few invalid surveys that were erroneously retained in the analysis, the number of such surveys is likely very small and likely to have minimal impact on reported results.

Raw survey data were abstracted from the REDCap database. Data cleaning and recording was managed using Excel. Analyses were performed using R, version 4.0.4. The number of respondents from each primary workplace was highly unbalanced, with more responses coming from acute care. Since respondents had the option of indicating that they did not experience any given barrier, denominators for percentages varied question to question. For the ratings of barriers, affirmative responses (i.e., moderately/greatly agree) were grouped together. We computed the proportion of respondents that agreed each listed factor was a barrier, post-stratified by age, job category and primary workplace, as well as differences within the above health care groups. While we report percentages post-stratified by age, we focused interpretation of results based on job category and primary workplace.

Responses to survey questions that were deemed most relevant to LTC/AL by stakeholders prior to reviewing results were compared to acute care responses. Permutation-based Monte Carlo tests for difference of proportions were performed using 999 random permutations for each comparison, as they tend to perform better than the usual Z-test for difference of proportions when group sizes are unbalanced [15]. Further, since there are several hypothesis tests performed from the same data set, the Benjamini-Hochberg procedure was used to adjust for multiple comparisons [15]. Accordingly, using a q-value of 0.05, tests with false discovery rate (FDR) less than 0.05 are considered statistically significant.

Thematic analysis of the qualitative survey responses was conducted independently by two members of the study team to summarize these responses, based on the frequency of specific terms used in the open text responses. Discrepancies between the two reviewers were adjudicated by a third member of the study team. Where applicable, qualitative responses were reclassified to a pre-specified recommendation or suggestion.

### Ethics approval

Based on the Provincial Health Services Authority Project Sorting Tool, [16] this project was determined to be a quality improvement intervention and involved minimal risk to participants. To address privacy concerns, no personally identifiable information was collected, and the final survey was reviewed by a privacy officer.

## Results

### Demographics

There were 3143 survey responses obtained through the REDCap online survey, of which 3110 gave consent to participate (participation rate 99.0%) and 2755 completed the survey with a timestamp (completion rate 88.6%). Of those 2755 completed surveys, 2488 were from eligible participants and were included in the final analyses. For the purposes of this paper, we focused on 896 respondents whose primary workplace was in acute care and 441 whose primary workplace was in LTC/AL. The remaining 1,151 participants worked in other health care settings. Responses were received from every health authority in British Columbia. At the time of the survey, there were over 120,000 employees of health authorities in BC and over 16,000 medical staff, although not all would have been eligible to participate. Overall in British Columbia, approximately 48% of HCWs work in acute care and 17% work in LTC/AL [17].

Participant demographics for the 1337 respondents working in acute care (67.0%) and LTC/AL (33.0%) are shown in Table 1.

**Table 1:** Respondent demographics

		Primary Workplace (% of workplace group)			
**Characteristic**	Full Cohort	Acute Care	LTC/AL	**Total analysis group (%)**	
Sample Size (% of total analysis group)	2488	896 (67.0)	441 (33.0)	1337 (100)	
**Gender** ^*^					
Female	1354	654 (73.0)	190 (43.1)	844 (63.1)	
Male	1035	222 (24.8)	240 (54.4)	462 (34.6)	
Other	99	8 (0.9)	8 (1.8)	16 ( 1.2)	
**Age Category**					
Less than 30 years	885	268 (30.0)	180 (40.8)	448 (33.5)	
30–39 years	903	290 (32.4)	163 (37.0)	453 (33.9)	
40–49 years	399	183 (20.4)	53 (12.0)	236 (17.7)	
50–59 years	229	113 (12.6)	32 (7.3)	145 (10.8)	
60 years or older	72	42 (4.7)	13 (2.9)	55 (4.1)	
**Years Worked in Health Care**					
5 or fewer	902	333 (37.2)	185 (42.0)	518 (29.9)	
6–10	816	205 (22.9)	156 (35.4)	361 (27.0)	
11–15	343	128 (14.3)	43 (9.8)	171 (12.8)	
16 or more	427	230 (25.7)	57 (12.9)	287 (21.5)	
**Job Category**					
Nursing	750	470 (52.5)	90 (20.4)	560 (41.9)	
Clinical support	684	328 (36.6)	111 (25.2)	439 (32.8)	
Paramedic	95	5 (0.6)	21 (4.8)	26 (1.9)	
Physician and other providers	61	19 (2.1)	7 (1.6)	26 (1.9)	
Other	896	74 (8.3)	212 (48.1)	286 (21.4)	

*15 respondents (12 in Acute Care, 3 in LTC/AL) preferred to not report their gender

### IPAC Knowledge

Acute care and LTC/AL respondents who did not work in IPAC (n = 771) were surveyed on their IPAC knowledge (Table 2). There were no significant differences between respondents in acute care vs. LTC/AL (FDR > 0.05) for all statistical tests performed and results were in line with those found among the full non-IPAC cohort (n = 1130). Overall, only 82% correctly indicated that alcohol-based hand sanitizer effectively kills the SARS-CoV-2 virus. Although 97% of all participants reported that they knew how to properly don and doff personal protective equipment (PPE), only 54% felt confident that they knew what PPE should be used for patients with suspected or confirmed COVID-19 (not including aerosol-generating medical procedures) and 16% missed at least one required component (gown, goggles, gloves, and/or mask) when asked to select what PPE was indicated. During the study period, provincial PPE guidelines changed, [18] and so selection of at least one of medical mask or N95 respirator was deemed correct. However, 9 respondents (1.2%) in acute care and LTC/AL chose neither medical mask nor N95 respirator or equivalent.

**Table 2:** Percentage of 771 non-IPAC respondents who answered IPAC knowledge questions affirmatively/correctly. Overall results for full cohort of 1130 non-IPAC responses included for reference

Population	Number of respondents (min, max)^*^	Believes alcohol-based sanitizer kills SARS-CoV-2 (%)	Knows how to don/doff PPE properly (%)	Believes they know correct PPE to use^**^ (%)	Chose correct PPE for suspected or confirmed COVID-19 patients %
Acute Care	(658, 663)	81	98	60	90
LTC/Assisted Living	(107, 108)	86	97	56	84
Overall	(1119, 1129)	82	97	54	84

*Number of respondents varied by question. **No statistical test performed

To further understand how HCWs obtain IPAC knowledge, non-IPAC respondents were asked to select which sources of information they accessed throughout the pandemic. Again, there were no statistically significant differences in the sources of IPAC information accessed by HCWs in acute care vs. LTC/AL (FDR > 0.05) for all comparisons and results aligned with that found among all 1130 non-IPAC respondents. Overall, information and guidance provided by one’s own health authority was the most commonly used resource (80%), followed by online education (60%), in person (58%), and own online searches (41%).

### Barriers to IPAC Practices

#### Perception

Of the non-IPAC respondents, perceptions of IPAC practices were generally favourable (Table 3), with a strong majority (just over 90%) believing that they prevent transmission in the workplace. However, compared to HCWs in acute care, more LTC/AL respondents felt that following IPAC guidance was of lower priority compared to other tasks (29.1% vs. 14.7%, FDR = 0.001) and that it was not their responsibility (28.0% vs. 11.2%, FDR = 0.001). Slightly less than 50% of respondents agreed that the risk of COVID-19 was low in the workplace.

**Table 3:** Percentage of respondents who moderately/greatly agreed that each COVID-19 perception factor affected their willingness to follow IPAC practices, among the 771 non-IPAC respondents. Overall results for full cohort of 1130 non-IPAC responses included for reference

Population	Number of respondents (min, max)*	IPAC practices prevent transmission of COVID-19 in workplace (%)	Other tasks/work have higher priority than IPAC practices (%)	Not my responsibility to ensure IPAC practices for COVID-19 are implemented (%)	Risk of COVID-19 is low in workplace (%)
Acute Care	(639, 660)	93	15	11	41
LTC/AL	(103, 107)	92	29	28	46
Overall	(1087, 1123)	92	20	17	45

*Respondents who experienced the barrier and provided a rating varied for each factor

### Guidance and Communication

Among all participants, the extent to which communication-related barriers affected ability to follow IPAC practices was mixed (Table 4). Significantly higher percentages of acute care respondents reported that frequent changes (55.7% vs. 47.0%, FDR = 0.004) and confusing messaging in IPAC guidelines (56.2% vs. 46.0%, FDR = 0.001) affected IPAC adherence compared to LTC/AL respondents. However, LTC/AL respondents were more likely to report contradictions in IPAC guidance as a barrier (FDR = 0.054).

**Table 4:** Percentage of respondents who moderately/greatly agreed that each COVID-19 guidance and communication factor affected their ability to follow IPAC practices among 1337 respondents. Overall results for full cohort of 2488 respondents included for reference

Population	Number of respondents (min, max)*	Frequent changes in IPAC guidance/ recommendations (%)	Confusing messages about IPAC practices within/from my workplace (%)	Contradictions in IPAC guidance between my workplace and other sources (%)
Acute Care	(834, 862)	56	57	48
LTC/AL	(415, 426)	48	46	56
Overall	(1672, 1733)	51	53	50

*Respondents who experienced the barrier and provided a rating varied for each factor

### Infrastructure

More LTC/AL respondents experienced challenges with limited supplies for PPE (49.0% vs. 33.6%, FDR = 0.001), hand hygiene products (42.2% vs. 28.8%, FDR = 0.001), and cleaning/disinfection products (44.1% vs. 30.3%, FDR = 0.001) compared to those in acute care (Table 5). However, limited physical space was the most commonly experienced barrier. Both acute and LTC/AL respondents reported physical space-related challenges with limited dining room availability and multi-bed patient rooms affecting IPAC practice. A higher percentage of acute care respondents reported limited staff room capacity as an experienced barrier (66.1% vs. 48.8%, FDR = 0.001), whereas wandering patients was a barrier cited more often in the LTC/AL setting (57.7% vs. 41.0%, FDR = 0.001).

**Table 5:** Percentage of respondents who moderately/greatly agreed that each infrastructure factor affected their ability to follow IPAC practices among all 1337 respondents. Percentages for the overall cohort of 2488 respondents is also reported for reference

Population	Number of respondents (min, max)*	Limited PPE (%)	Limited hand hygiene products (%)	Limited cleaning products (%)	Limited space capacity in staff rooms (%)	Prolonged close proximity to patients (%)	Multi-bed rooms (%)	Limited space capacity in patient dining spaces (%)	Cluttered areas (%)	Limited dedicated clean space (%)	Wandering patients (%)
Acute Care	(462, 861)	34	29	30	66	51	56	45	47	39	41
LTC/Assisted Living	(408, 422)	50	42	45	49	47	51	51	44	45	58
Overall	(1784, 2369)	44	36	39	57	48	51	46	46	42	49

*Respondents who experienced the barrier and provided a rating varied for each factor

### Front Line Work Environment

Limited staff for covering absences was the most commonly cited barrier to IPAC practices among those working in acute care settings (70%) and overall (58%) (Table 6). A higher percentage of LTC/AL respondents reported deficits in IPAC leadership support (46.2% vs. 38.9%, FDR = 0.012), IPAC education and training (46.9% vs. 32.0%, FDR = 0.001), and patient care knowledge for managing COVID-19 infections (46.6% vs. 36.0%, FDR = 0.001) compared to acute care. LTC/AL respondents also more frequently reported having limited time and/or being too busy to follow IPAC practices (50.5% vs. 29.9%, FDR = 0.001). However, acute care respondents more frequently reported experiencing burnout or fatigue (55.9% vs. 46.6%, FDR = 0.002) and staff limitations to cover sick leave absences (69.3% vs. 55.3%, FDR = 0.001).

**Table 6:** Percentage of respondents who moderately/greatly agreed that each front line work environment factor affected their ability to follow IPAC practices among all 1337 respondents. Percentages for the overall cohort of 2488 respondents is also reported for reference

Population	Number of respondents (min, max)*	Limited time (%)	Burnout/fatigue (%)	Tired of cleaning hands (%)	Concerns for hand skin (%)	Tired of wearing PPE (%)	PPE affects function (%)	Concerns for skin (%)	Limited cleaning staff (%)	Limited staff for leave (%)	Limited IPAC staff (%)	Limited IPAC leadership (%)	Limited IPAC communication (%)	Limited IPAC training (%)	Limited outbreak knowledge (%)	Ltd patient management knowledge (%)
Acute Care	(784, 878)	30	56	30	42	49	46	40	47	70	41	39	41	32	39	36
LTC/AL	(410, 427)	51	47	43	45	48	51	45	48	56	47	47	44	47	44	47
Overall	(2229, 2398)	41	49	37	44	48	46	43	46	58	42	41	42	39	41	41

*Respondents who experienced the barrier and provided a rating varied for each factor

### Suggestions for improvement

The most popular categories of suggestions for improvement in both acute care and LTC/AL were related to increasing IPAC leadership and support, and addressing communication barriers (Table 7). The suggestions aligned with the identified barriers, with LTC/AL respondents most frequently indicating more IPAC leadership and support (61%) and acute care respondents stating more frequent and clear communication (63%) was needed.

**Table 7:** Suggestions selected or written by respondents to improve ability to follow IPAC practices

		Suggested Improvements (%)				
Population	Number of respondents	Improved access to PPE and hand hygiene	More IPAC leadership, support	More frequent and clear communication	More in-person training, education	Other suggestions*
Acute Care	868	57	57	63	36	7
LTC/AL	433	44	61	46	31	2
Overall	2436	47	60	56	32	4

*Staffing and workload issues included with “Other suggestions”

## Discussion

Overall, this province-wide survey found that certain barriers to HCWs’ adherence to IPAC practices were different in LTC/AL settings compared to acute care during the COVID-19 pandemic. LTC/AL respondents were more likely to perceive IPAC as a low priority and not their responsibility; have limited access to PPE, hand hygiene, and cleaning and disinfection products; be too busy to follow IPAC practices; and have limited IPAC leadership and education in their settings. Acute care respondents were more likely to report confusing IPAC messages, frequent changes to guidance, burnout/fatigue, and limited staff available to cover sick leaves.

LTC/AL was a high risk setting for transmission of infections even before the pandemic due to multiple factors. Although some are non-modifiable, such as the age and comorbidities of residents or the need for staff to provide frequent close personal care (e.g. feeding, bathing), other contributors can be targets for quality improvement. For example, there were disparities between IPAC programs in LTC/AL compared to acute care prior to pandemic, with LTC/AL facilities having fewer IPAC staff, IPAC staff with less training, and less involvement of IPAC staff with everyday operations [6, 7, 19]. AL facilities in particular were unlikely to have robust IPAC programs, policies, or training at the start of the pandemic [20, 21]. During the pandemic, studies showed that lower scores on an assessment of IPAC policies and practices were associated with increased severity of COVID-19 outbreaks in LTC facilities [22, 23]. This is consistent with our finding that LTC/AL staff were more likely to report gaps in IPAC programs as barriers, including limited access to supplies and deficiencies in IPAC leadership/education.

Staffing of LTC/AL facilities has been associated with COVID-19 cases and outbreaks during the pandemic. In one of the early reports of a LTC facility outbreak in the United States, transmission was likely exacerbated by staff working in multiple facilities, in addition to frequent patient transfers between acute and LTC facilities [24]. Higher pre-pandemic staffing levels have also been shown to correlate with reduced risk of COVID-19 cases and outbreaks in LTC/AL settings [25, 26]. Limited staff for covering sick leave absences was among the most commonly reported barriers by LTC/AL respondents in our survey. However, those in LTC/AL were less likely to experience this as a barrier compared to acute care, perhaps related to province-wide policies implemented early in the pandemic to minimize the risk of transmission in LTC/AL facilities [27].

Although many studies have assessed facilitators and barriers to IPAC practices prior to and during the pandemic, few have examined LTC/AL settings. A systematic review focusing on IPAC guidelines for respiratory infections prior to the pandemic did not include any studies conducted in LTC/AL [28]. Regardless, there were some similarities to our survey, such as lack of training and conflicting guidance being among the most commonly reported barriers in the systematic review. One Canadian needs assessment survey of IPAC professionals conducted prior to the pandemic found that motivation of LTC/AL stakeholders to participate in IPAC initiatives was the barrier most commonly selected by respondents [6]. Consistent with this finding, LTC/AL respondents to our survey were more likely than acute care respondents to perceive IPAC as a low priority and not their responsibility. Implementation of IPAC programs using a front line ownership approach, focusing on empowerment of front line staff to address IPAC challenges in their workplaces, may be beneficial to address this particular barrier [29].

This study, to our knowledge, is the first Canadian survey to comprehensively assess barriers to IPAC practices among HCWs working in LTC/AL and comparing to acute care during the COVID-19 pandemic. However, there are some limitations. The sample of survey responses might not be fully representative of HCWs in those settings and may underestimate barriers. For example, HCWs who are unable to follow IPAC practices may also be unable to complete a survey, due to excessive workload, burnout, or limited computer access. Moreover, in our respondent groups, there was a higher proportion of IPAC vs. non-IPAC professionals working in acute care compared to LTC/AL, which may have affected the perspectives regarding relevant barriers. Second, the overall response rate was low given that there are over 120,000 employees of health authorities in BC and over 16,000 medical staff. However, not all of those would have been eligible to participate since the numbers include administrative and other non-clinical jobs. Further, this survey was conducted over one year into the pandemic, and responses may have been impacted by the ongoing promotion of IPAC practices both in the workplace and in public settings. Finally, only HCWs at health authority operated LTC/AL facilities were eligible to participate, so the results may not apply to privately owned facilities.

This project has identified areas for future research. Additional qualitative research to better understand the most important barriers from the front line worker experience, specifically targeting LTC/AL settings, would be beneficial, particularly focusing on groups that may have been under-represented among respondents, such as environmental services staff who may not have regular access to computers.

## Conclusions

In summary, our findings highlight major barriers to IPAC practices in LTC/AL compared to acute care settings in British Columbia. Improvement efforts should focus on strengthening IPAC programs in LTC/AL, particularly enhanced staffing/leadership, increased training and education, and improving access to PPE, hand hygiene supplies, and cleaning products. LTC/AL facilities may further benefit from the use of front line ownership approach to implement these strategies as opposed to top-down decisions or strategies that do not take the realities of front line work into account. Addressing the gaps identified by this survey must be a priority in order to protect the LTC/AL population most at risk of severe outcomes due to COVID-19.

### Electronic supplementary material

Below is the link to the electronic supplementary material.

**Supplementary Material 1**: Full Survey Questionnaire

## Data Availability

The data that support the findings of this study are not openly available due to reasons of sensitivity and are available from the corresponding author upon reasonable request. Data are located in controlled access data storage at the Provincial Health Services Authority.
