# Supplementary material for: Barriers to infection prevention and control in long-term care/assisted living settings in British Columbia during the COVID-19 pandemic: a cross-sectional survey
Source: Antimicrob Resist Infect Control. 2023 Aug 30;12:84. doi: 10.1186/s13756-023-01292-2 (PMC10469816; doi:10.1186/s13756-023-01292-2)
Supplement: Supplementary file 1 — Supplementary Material 1: Full Survey Questionnaire [file 13756_2023_1292_MOESM1_ESM.pdf]

Barriers to health care staff's ability to follow IPAC practice in BC during the COVID-19 pandemic

DescriptionThis initiative is a collaborative quality improvement project run by infection prevention and control (IPAC) and Workplace Health & Safety professionals from across British Columbia (BC), with affiliations to the University of British Columbia (UBC), the Provincial Infection Control Network of BC (PICNet), Infection Prevention & Control Epidemiologists of British Columbia (ICE-BC) and multiple health authorities.

Principal Investigator: Dr. Jocelyn Srigley - Clinical Assistant Professor, University of British Columbia; Medical Microbiologist and IPAC Medical Lead, BC Children's Hospital and BC Women's Hospital + Health Centre; Investigator, BC Children's Research Institute

Project GoalsWe aim to identify the barriers affecting health care staff ability to follow IPAC practices during the COVID-19 pandemic in BC. This survey is targeted towards individuals working in acute care, long-term care, assisted living, outpatient setting(s), pre-hospital care and/or home care, who hold one of the following roles:

Staff providing direct patient care or working in the patient environment (e.g. entering patient rooms, face-to-face interaction with patients)

IPAC professionals who interact with direct care providers

This work will serve to inform future IPAC practices and interventions for health authorities across the province, with the goal of preventing transmission of COVID-19 as well as future emerging infections.

Participant Involvement & ConsentEstimated survey time: 10 mins

Completion of the survey is voluntary. The deadline to complete this survey is September 10th, 2021. If you do the survey, you are consenting (agreeing) to participate.

The results of this survey are confidential. The personal information you will be asked to provide will be your age, gender identity, and opinions.

Your personal information is protected by our privacy law in BC. This law is called the Freedom of Information and Protection of Privacy Act (FIPPA). We are collecting your information under section 26 (e) of FIPPA.

We hope that collecting this information can lead to positive change which includes revealing inequities and relationships between demographic categories. The study team respects the values and culture of all patients.

Only the study team and the technical support team at Provincial Health Services Authority (PHSA) will access your information. When results are reported, presented, or published the project team will remove any information that could identify you or anyone else.

The team may use a quote from your comments if provided.

Survey data will be stored securely at PHSA for at least 5 years. Survey results will be available on www.picnet.ca after analyses are complete.

To thank you for your time, you can submit your health authority email address to be entered in a random draw to win one of 6 x $50 Amazon gift cards upon completion of the survey. This is optional. Your email address will be kept separate from your survey responses and your survey answers will remain confidential. If you do not have a health authority email address, please contact Brooke Cheng (brooke.cheng@cw.bc.ca) to be entered into the draw.

If you have any questions, please contact: Brooke Cheng - Research Assistant (brooke.cheng@cw.bc.ca).

We are grateful for your participation!

Consent

I have read and understand this form. I voluntarily consent to the study team collecting, using and disclosing the information I provide.	◯ I consent (proceed to survey)<br>◯ I do not consent (exit survey)

Eligibility & Occupational Information

Are you an Infection Prevention and Control Professional (e.g. infection control practitioner or infection control physician)?	◯ Yes<br>◯ No
Do you interact with direct care providers (e.g. front-line staff)?	◯ Yes<br>◯ No
Do you provide direct patient care or work in the patient environment (e.g. entering patient rooms, face-to-face interaction with patients)?	◯ Yes<br>◯ No
Please select the health authority that operates the facility where you spent most of your work time since the COVID-19 pandemic:	◯ First Nations Health Authority<br>◯ Fraser Health<br>◯ Interior Health<br>◯ Island Health<br>◯ Northern Health<br>◯ Providence Health Care<br>◯ Provincial Health Services Authority<br>◯ Vancouver Coastal Health<br>◯ None of the above
Please select the category that best describes your profession:	◯ Allied Health professional (e.g. pharmacist, radiation therapist, laboratory staff)<br>◯ Environmental services (housekeeping)<br>◯ Facilities maintenance staff<br>◯ Food services<br>◯ Nurse<br>◯ Paramedic<br>◯ Physician<br>◯ Other
If other, please specify your role:	__________________________________
Do you work in a designated COVID-19 unit or zone?	◯ Yes<br>◯ No<br>◯ I work on both COVID-19 unit(s) and other unit(s) (e.g. throughout the hospital)
How many years have you worked in a health care setting?	◯ Less than 1 year<br>◯ 1-5 years<br>◯ 6-10 years<br>◯ 11-15 years<br>◯ 16-20 years<br>◯ More than 20 years
How many years have you been working in infection prevention and control (IPAC)?	◯ Less than 1 year<br>◯ 1-5 years<br>◯ 6-10 years<br>◯ 11-15 years<br>◯ 16-20 years<br>◯ More than 20 years

Which types of health care setting(s) do you work in? (Select all that apply.)	☐ Acute care<br>☐ Long-term care and/or Assisted Living<br>☐ Outpatient/Community Clinic (i.e. standalone facility)<br>☐ Home care visits<br>☐ Mental health<br>☐ Pre-hospital care (i.e. BC Emergency Services)<br>☐ Other
If other, please specify:	__________________________________
Of the health care settings you selected in the previous question, which is your primary workplace?<br><br>(I.e. The workplace where you spend the majority of your work hours.)	◯ Acute care<br>◯ Long-term care and/or Assisted Living<br>◯ Outpatient/Community Clinic (i.e. standalone facility)<br>◯ Home care visits<br>◯ Mental health<br>◯ Pre-hospital care (i.e. BC Emergency Services)<br>◯ Other
If other, please specify:	__________________________________
Do you directly provide infection prevention and control (IPAC) education/training related to COVID-19 to other health care workers or staff?	◯ Yes<br>◯ No
Do you have previous experience working in health care settings during a period of epidemic or pandemic preparedness or response? (e.g. SARS [SARS-CoV-1], Swine Flu [H1N1], Ebola)	◯ Yes<br>◯ No

Demographic Information

We hope that collecting this information can lead to positive changes which includes revealing inequities and relationships between demographic categories. The study team respects the values and culture of all patients.

Please select your age group:

◯ < 20 years old
◯ 20-29 years old
◯ 30-39 years old
◯ 40-49 years old
◯ 50-59 years old
◯ 60-69 years old
◯ 70+ years old

Which gender identity or identities do you use to describe yourself? (Select all that apply.)

☐ Male
☐ Female
☐ Transgender
☐ Non-binary
☐ Other
☐ Prefer not to answer

If other, please specify:

____

IPAC Knowledge & Learning Assessment

During the COVID-19 pandemic, where have you gotten information about infection prevention and control (IPAC) practices related to COVID-19 in your health care facility? (Select all that apply.)	☐ In-person education/training/discussion<br>☐ Online education/training/discussion<br>☐ Received information/guidance/resources from my health authority<br>☐ Searched out information/guidance online on my own<br>☐ I have not used/accessed any of these sources
Which online source(s) did you access for COVID-19 information? (Select all that apply.)	☐ British Columbia Centre for Disease Control (BCCDC)<br>☐ Public Health Agency of Canada<br>☐ BC Ministry of Health<br>☐ World Health Organization (WHO) websites<br>☐ United States Centers for Disease Control and Prevention (US CDC)<br>☐ My own health authority website<br>☐ Other
If other, please specify:	__________________________________
What personal protective equipment (PPE) should be used when providing general care (no aerosol-generating medical procedures) to patients confirmed to have or under investigation for COVID-19? (Select all that apply.)	☐ Gown<br>☐ Gloves<br>☐ Medical mask<br>☐ N95 respirator or equivalent<br>☐ Eye protection (e.g. goggles, face shield)
Do you know how to properly use PPE (e.g., don/put-on, doff/take-off)?	○ Yes<br>○ No<br>○ I don't know
Is alcohol-based hand sanitizer effective for killing the SARS-CoV-2 virus?	○ Yes<br>○ No<br>○ I don't know

BARRIER ASSESSMENT

These questions will ask you to rate how the factors below have affected your ability to follow COVID-19 infection prevention and control (IPAC) practices in your primary workplace.

Section A: Perception

How strongly do you agree or disagree with the following statements?

	Strongly disagree	Disagree	Agree	Strongly agree	Prefer not to answer
The risk of COVID-19 is low in my workplace	○	○	○	○	○
Following IPAC practices will prevent transmission of COVID-19 in my workplace	○	○	○	○	○
I have other tasks/work that are higher priority than IPAC practices	○	○	○	○	○
It is not my responsibility to ensure that IPAC practices for COVID-19 are implemented	○	○	○	○	○

Section B: Guidance and Communication

In your primary workplace, how much did the factors below affect your ability to follow IPAC practices during the COVID-19 pandemic?

	Did not experience this/Not applicable	Not at all	Slightly	Moderately	Greatly	Prefer not to answer
Frequent changes in IPAC guidance/recommendations	◯	◯	◯	◯	◯	◯
Confusing messages about IPAC practices within/from my workplace	◯	◯	◯	◯	◯	◯
Contradictions in IPAC guidance between my workplace and other sources (e.g. provincial, national or international guidance)	◯	◯	◯	◯	◯	◯

Section C: Infrastructure
In your primary workplace, how much did the factors below affect your ability to follow IPAC practices during the COVID-19 pandemic?

NOTE: The term "patient" will refer to patients, clients and residents.

	Did not experience this/Not applicable	Not at all	Slightly	Moderately	Greatly	Prefer not to answer
Limited availability of personal protective equipment (PPE) (e.g. mask, gown, gloves, etc.)	○	○	○	○	○	○
Limited availability of hand hygiene products (e.g. soap or alcohol-based hand sanitizer)	○	○	○	○	○	○
Limited availability of cleaning/disinfecting products (e.g. cleaning wipes)	○	○	○	○	○	○
Limited space capacity of staff rooms	○	○	○	○	○	○
Close proximity to patients for long time periods (e.g. bathing, physical examinations, etc.)	○	○	○	○	○	○
Multi-bed patient rooms	○	○	○	○	○	○
Limited space capacity of patient dining rooms	○	○	○	○	○	○
Clutter in patient areas	○	○	○	○	○	○
Limited dedicated clean and dirty areas/rooms (e.g. for garbage, laundry, clean linens)	○	○	○	○	○	○
Wandering patients	○	○	○	○	○	○

Section D: Front-Line Work Environment
In your primary workplace, how much did the factors below affect your ability to follow IPAC practices during the COVID-19 pandemic?

NOTE: The term "patient" will refer to patients, clients and residents.

	Did not experience this/Not applicable	Not at all	Slightly	Moderately	Greatly	Prefer not to answer
Limited time/too busy to follow IPAC practices	○	○	○	○	○	○
Feeling burnout/fatigue	○	○	○	○	○	○
Feeling tired of cleaning hands so frequently	○	○	○	○	○	○
Concerns with skin condition due to cleaning hands frequently	○	○	○	○	○	○
Feeling tired of wearing COVID-19-related PPE (e.g. mask, eye protection) all the time	○	○	○	○	○	○
Difficulty with normal function (e.g. vision, mobility, dexterity) due to wearing PPE	○	○	○	○	○	○
Concerns with skin condition due to frequent PPE use	○	○	○	○	○	○
Limited cleaning staff to perform enhanced cleaning	○	○	○	○	○	○
Not enough staffing to cover sick leave absences	○	○	○	○	○	○
Limited IPAC staff availability/experience	○	○	○	○	○	○
Limited leadership support for IPAC practices	○	○	○	○	○	○
Not enough communication between IPAC team and front-line health care workers	○	○	○	○	○	○
Not enough education/training on recommended IPAC practices	○	○	○	○	○	○
Limited knowledge of how to manage a COVID-19 outbreak	○	○	○	○	○	○
Limited knowledge of IPAC requirements to manage COVID-19-positive patients	○	○	○	○	○	○

BARRIER ASSESSMENT
These questions will ask you to rate various factors and their effects on staff ability to follow COVID-19 IPAC practices.

Section A: Guidance and Communication
In your primary workplace, how much did the factors below affect the ability of staff to follow IPAC practices during the COVID-19 pandemic?

	Did not experience this/Not applicable	Not at all	Slightly	Moderately	Greatly	Prefer not to answer
Frequent changes in IPAC guidance/recommendations	○	○	○	○	○	○
Confusing messages about IPAC practices within/from my workplace	○	○	○	○	○	○
Contradictions in IPAC guidance between my workplace and other sources (e.g. provincial, national or international guidance)	○	○	○	○	○	○

Section B: Infrastructure
In your primary workplace, how much did the factors below affect the ability of staff to follow IPAC practices during the COVID-19 pandemic?

NOTE: The term "patient" will refer to patients, clients and residents.

	Did not experience this/Not applicable	Not at all	Slightly	Moderately	Greatly	Prefer not to answer
Limited availability of personal protective equipment (PPE) (e.g. mask, gown, gloves, etc.)	○	○	○	○	○	○
Limited availability of hand hygiene products (e.g. soap or alcohol-based hand sanitizer)	○	○	○	○	○	○
Limited availability of cleaning/disinfecting products (e.g. cleaning wipes)	○	○	○	○	○	○
Limited space capacity of staff rooms	○	○	○	○	○	○
Close proximity to patients for long time periods (e.g. bathing, physical examinations, etc.)	○	○	○	○	○	○
Multi-bed patient rooms	○	○	○	○	○	○
Limited space capacity of patient dining rooms	○	○	○	○	○	○
Clutter in patient areas	○	○	○	○	○	○
Limited dedicated clean and dirty areas/rooms (e.g. for garbage, laundry, clean linens)	○	○	○	○	○	○
Wandering patients	○	○	○	○	○	○

Section C: Front-Line Work Environment
In your primary workplace, how much did the factors below affect the ability of staff to follow IPAC practices during the COVID-19 pandemic?

NOTE: The term "patient" will refer to patients, clients and residents.

	Did not experience this/Not applicable	Not at all	Slightly	Moderately	Greatly	Prefer not to answer
Limited time/too busy to follow IPAC practices	○	○	○	○	○	○
Feeling burnout/fatigue	○	○	○	○	○	○
Feeling tired of cleaning hands so frequently	○	○	○	○	○	○
Concerns with skin condition due to cleaning hands frequently	○	○	○	○	○	○
Feeling tired of wearing COVID-19-related PPE (e.g. mask, eye protection) all the time	○	○	○	○	○	○
Difficulty with normal function (e.g. vision, mobility, dexterity) due to wearing PPE	○	○	○	○	○	○
Concerns with skin condition due to frequent PPE use	○	○	○	○	○	○
Limited cleaning staff to perform enhanced cleaning	○	○	○	○	○	○
Not enough staffing to cover sick leave absences	○	○	○	○	○	○
Limited IPAC staff availability/experience	○	○	○	○	○	○
Limited leadership support for IPAC practices	○	○	○	○	○	○
Not enough communication between IPAC team and front-line health care workers	○	○	○	○	○	○
Not enough education/training on recommended IPAC practices	○	○	○	○	○	○
Limited knowledge of how to manage a COVID-19 outbreak	○	○	○	○	○	○
Limited knowledge of IPAC requirements to manage COVID-19-positive patients	○	○	○	○	○	○

Ability to Follow IPAC Practices

At the BEGINNING of the COVID-19 pandemic (Jan-May 2020), how often were you able to follow IPAC practices (e.g. hand hygiene, PPE donning/doffing, cleaning/disinfecting equipment, etc.) in your primary workplace?

(Reminder: This information is being collected confidentially.)

Never ______________________________ Always

(Place a mark on the scale above)

During the MOST RECENT wave of the COVID-19 pandemic (Mar-Jun 2021), how often were you able to follow IPAC practices (e.g. hand hygiene, PPE donning/doffing, cleaning/disinfecting equipment, etc.) in your primary workplace?

(Reminder: This information is being collected confidentially.)

Never ______________________________ Always

(Place a mark on the scale above)

To what extent do you think your ability to follow IPAC practices has changed during the COVID-19 pandemic?

- ◯ Much worse
- ◯ Somewhat worse
- ◯ Stayed the same
- ◯ Somewhat better
- ◯ Much better

Recommendations & Suggestions

Which three areas do you think could most improve your ability to follow IPAC practices in health care settings during the COVID-19 pandemic? (Select all that apply.)

- ☐ Improved access to PPE
- ☐ Improved access to hand hygiene products
- ☐ More leadership support
- ☐ More support from Infection Prevention and Control Professionals (e.g. education, consultation, resources, etc.)
- ☐ More in-person training and education
- ☐ More frequent communication about required IPAC practices (e.g. email, in-person meetings, in-person training, etc.)
- ☐ More clear communication about required IPAC practices
- ☐ Other suggestions (use box provided)
- ☐ Prefer not to answer

If other, please specify:

____

From your viewpoint, please provide the three most important suggestions/recommendations to help front-line health care workers better follow IPAC practices during the COVID-19 pandemic.

- ☐ Improved access to PPE
- ☐ Improved access to hand hygiene products
- ☐ More leadership support
- ☐ More Infection Prevention and Control Professionals
- ☐ More in-person training and education
- ☐ More frequent communication about required IPAC practices (e.g. email, in-person meetings, in-person training, etc.)
- ☐ More clear communication about required IPAC practices
- ☐ Other suggestions (use box provided)
- ☐ Prefer not to answer

If other, please specify:

____
